# Supplementary material for: Hybrid Models and Biological Model Reduction with PyDSTool
Source: PLoS Comput Biol. 2012 Aug 9;8(8):e1002628. doi: 10.1371/journal.pcbi.1002628 (PMC3415397; doi:10.1371/journal.pcbi.1002628)
Supplement: Text S4 — Complete source code for the PyDSTool package (version 0.88.120504). Includes API documentation and help files linking to web pages. This file is identical to the current public release on Sourceforge.net. (ZIP) [file pcbi.1002628.s004.zip › PyDSTool/html/PyDSTool.Toolbox.optimizers.optimizer.optimizer-pysrc.html]

xml version="1.0" encoding="ascii"?


PyDSTool.Toolbox.optimizers.optimizer.optimizer


| Home | Trees | Indices | Help | PyDSTool |
| --- | --- | --- | --- | --- |

Package PyDSTool ::
Package Toolbox ::
Package optimizers ::
Package optimizer ::
Module optimizer

[hide private]

[frames] | no frames]

# Source Code for Module PyDSTool.Toolbox.optimizers.optimizer.optimizer

```
 1   
 2  # Matthieu Brucher 
 3  # Last Change : 2007-08-23 21:53 
 4   
 5  """ 
 6  The core optimizer from which every other optimizer is derived 
 7  """ 
 8   


9 -class Optimizer(object):


10    """ 
11    The simple optimizer class 
12    This class lacks some intel that must be populated/implemented in the subclasses : 
13      - currentValues is a list of the 2 last cost values computed with the function 
14      - currentParameters is a list of the 2 last parameters used for the computation of currentValues 
15      - the iterate function that does the real iteration loop 
16    """ 


17 -  def __init__(self, **kwargs):


18      """ 
19      Initialization of the optimizer, saves the function and the criterion to use 
20      Needs to have : 
21        - a function to optimize (function) 
22        - a criterion to stop the optimization (criterion) 
23      Can have : 
24        - a step modifier, a factor to modulate the step (stepSize = 1.) 
25        - a recorder that will be called with the factors used in one iteration step (record = self.recordHistory) 
26      """ 
27      # The global state of the optimizer, is passed to every sub module 
28      self.state = {} 
29   
30      self.state['iteration'] = 0 
31      self.optimized = False 
32   
33      if 'function' in kwargs: 
34        self.function = kwargs['function'] 
35      else: 
36        class Function(object): 
37          pass

38   
39        self.function = Function() 
40        self.function.__call__ = kwargs['fun'] 
41        self.function.__dict__.update(kwargs) 
42   
43      self.state['function'] = self.function 
44      self.criterion = kwargs['criterion'] 
45      self.recordHistory = kwargs.get('record', self.recordHistory) 
46      self.check_arguments()

47   


48 -  def optimize(self):


49      """ 
50      Does the optimization, call iterate and returns the optimal set of parameters 
51      """ 
52      if not self.optimized: 
53        self.iterate() # needed because we need a do while loop 
54        self.state['iteration'] += 1 
55        while(not self.criterion(self.state)): 
56          self.iterate() 
57          self.state['iteration'] += 1 
58   
59        self.optimized = True 
60   
61      return self.optimalPoint

62   


63 -  def recordHistory(self, **kwargs):


64      """ 
65      Function that does nothing, called for saving parameters in the iteration loop, if needed 
66      """ 
67      pass

68   


69 -  def iterate(self):


70      """ 
71      Does one iteration of the optimization 
72      Present here for readability 
73      """ 
74      NotImplemented

75   


76 -  def check_arguments(self):


77      """ 
78      Checks if the given arguments are correct 
79      """ 
80      if not hasattr(self.function, 'hessianvect'): 
81        self.function.hessianvect = lambda x, v: numpy.dot(self.function.hessian(x), v)

82
```

  


| Home | Trees | Indices | Help | PyDSTool |
| --- | --- | --- | --- | --- |

|  |  |
| --- | --- |
| Generated by Epydoc 3.0.1 on Fri May 4 15:24:18 2012 | http://epydoc.sourceforge.net |
